# Evaluation of Serum Creatinine Levels with Reference Change Value in Patients Receiving Colistin Treatment

**DOI:** 10.1093/labmed/lmad009

**Published:** 2023-10-26

**Authors:** Havva Yasemin Cinpolat, Sevil Alkan, Hatice Betul Altinisik, Dilek Ulker Cakir, Hamdi Oguzman

**Affiliations:** Department of Medical Biochemistry, Faculty of Medicine, Canakkale Onsekiz Mart University, Canakkale, Turkey; Department of Infectious Diseases, Faculty of Medicine, Canakkale Onsekiz Mart University, Canakkale, Turkey; Department of Anesthesiology and Reanimation, Faculty of Medicine, Canakkale Onsekiz Mart University, Canakkale, Turkey; Department of Medical Biochemistry, Faculty of Medicine, Canakkale Onsekiz Mart University, Canakkale, Turkey; Department of Medical Biochemistry, Faculty of Medicine, Hatay Mustafa Kemal University, Antakya, Hatay, Turkey

**Keywords:** biological variation, colistin, adverse effects, toxicity, clinical chemistry, reference values

## Abstract

**Objective:**

In this study, we aimed to evaluate the serum creatinine (SCr) levels with the reference change value (RCV) in patients receiving colistin treatment.

**Methods:**

We retrospectively recorded the SCr levels of 47 patients receiving colistin treatment before treatment and on days 3 and 7 after treatment. RCV was calculated with the asymmetrical RCV formula (*Z *= 1.64, *P *< .05). Percent (%) increase in the SCr results of the patients was compared with RCV and values exceeding RCV were regarded as statistically significant.

**Results:**

The RCV was calculated as 15.6% for SCr. Compared with pretreatment values, SCr value on day 3 was 32/47 and on day 7 it was 36/47; as these results exceeded RCV, they were considered statistically significant.

**Conclusion:**

Use of RCV in the interpretation of results between serial measurements will provide a more rapid and sensitive method when making decisions.

Population-based reference ranges are obtained from the distribution of the results of individuals with emphasis on different age groups, sex, time of sample collection, position of sample collection (supine or upright), smoking habits, pregnancy, etc, by using direct or indirect methods. When previous results for an individual cannot be obtained or if test results are being interpreted for the first time, these ranges become very useful.^1^ In instances where consecutive measurements are needed, the changes in results for a person can be due to the progression of disease, or it may as well be due to intra-individual biological variation (CVW) and analytical variation. In addition to population based reference ranges, using reference change values (RCVs) that include analytical variation and biological variation will increase the precision of interpretation of the results.^2^ By CVW with inter-individual biological variation (CVG), individuality index is calculated. If the individuality index is <0.6, population-based reference ranges would include only a limited number of subjects.^3,4^

Serum creatinine (SCr) is a cheap, accessible, and fast parameter for the assessment of nephrotoxicity, so it is a frequently preferred test. However, the individuality index calculated for SCr is 0.31 based on data obtained from the European Federation of Clinical Chemistry and Laboratory Medicine (EFLM) database, and it demonstrates high level of individuality.^5^ This means that the population-based reference range does not cover all the results. Although a patient’s results may be within the reference range, when a comparison is made between later and initial results of the same person, the difference can be significant. Thus, RCV gains further importance in routine patient follow-ups.^6^

Depending on the increase in SCr, RIFLE (Risk, Injury, Failure, Lost, End Stage Renal Disease), AKIN (Acute Kidney Injury Network) and KDIGO (Kidney Disease: Improving Global Outcomes) criteria are used in the classification of acute kidney injury (AKI).^7–9^ The RIFLE classification is based on a proportional increase in SCr or a decrease in glomerular filtration rate, whereas the AKIN classification is based on a proportional or an absolute increase in SCr and decrease in urine output. The KDIGO criteria was formed by combining with these 2 criteria.

Colistin is an antibiotic used in the 1960s that lost its place to other antibiotics with fewer side effects, as it was found to cause nephrotoxicity. During the last 10 to 15 years, with the emergence of multiple antibiotic resistance to Gram-negative bacteria, colistin use has increased.^10^

The most common and important side effect of colistin is nephrotoxicity. It develops within a week after initiation of treatment. This side effect is dose dependent and mostly reversible. Permanent kidney damage can rarely develop.^11^ The mechanism of nephrotoxicity for colistin is similar to its antibacterial effect. Colistin increases the membrane permeability of tubular cells. Increases in the permeability of anions, cations, and water result in the swelling and destruction of tubular cells. This then leads to acute tubular necrosis. The SCr levels of the person increase, and decreases in creatinine clearance, proteinuria, cylindruria, or oliguria may be seen^12,13^

In this study, we aimed to calculate the RCV for SCr measured in our laboratory, evaluate serial SCr measurements of patients receiving colistin treatment to demonstrate the development of nephrotoxicity with RCV, and investigate diagnostic accuracy of RCV by comparison with KDIGO criteria.

## Materials and Methods

The study was carried out retrospectively by obtaining data from medical reports at the university hospital between 2018 and 2021. Included subjects were over 18 years of age, had infections with Gram-negative bacteria that showed multiple antibiotic resistance, and had received colistin for 10 to 14 days at the dose that was prescribed according to their renal function (colistimethate sodium 150 mg intravenously [IV]; Kolistipol). Patients under 18 years of age, who were pregnant, had a history of rhabdomyolysis, received renal replacement therapy, had history of previous colistin use, used nephrotoxic medications other than colistin, or were given radiocontrast material were excluded. By performing electronic archive screening, SCr values before the start of colistin treatment and 3 and 7 days after and demographic data like age, sex, indication for administering colistin, and underlying diseases were recorded.

The approval of Canakkale Onsekiz Mart University Medical School Clinical Research Ethical Committee (date 09.06.2021 number 06-17) was obtained.

The population-based reference interval for SCr was defined as 0.70 to1.20 mg/dL for males and 0.50 to 0.90 mg/dL for females.

The increase in SCr more than ≥0.3 mg/dL within 48 hours or the increase in SCr more than 1.5-fold from the baseline was defined as colistin nephrotoxicity.^9^

SCr was measured with the kinetic alkaline picrate method on a cobas 8000 autoanalyzer (Roche Diagnostics). For calculating within-laboratory precision, 2-level internal quality control (Precicontrol 1 [lot No. 41011000] and Precicontrol 2 [lot No. 41003200]; Roche Diagnostics) material was used. The same operator measured each level of material for 20 testing days, 2 runs per testing day and 2 replicate measurements per run, and Westgard multirules were used to accept or reject these runs.^14^

### Statistical Analysis

The SPSS v.18.0 software was used for statistical analysis. The normality of data distribution was determined using the Shapiro-Wilk test. Continuous data that did not have a normal distribution was expressed as median (interquartile range), those with normal distribution as mean plus or minus standard deviation (SD), and categorical variables as proportions. The difference between SCr at different time points was compared by one-way analysis of variance with repeated measures and Bonferroni was used for post hoc analysis. Exact *P* value was given and *P* values of <.05 were considered as statistically significant.

Within-laboratory precision was calculated according to the Clinical and Laboratory Standards Institute (CLSI) document EP05:A3.^14^ The CVw was obtained by using the biological variation database published on the EFLM website.^5^ The RCV was calculated according to the asymmetrical RCV formula^15^:


$$\begin{array}{l} RCV=100\%*\left[exp(Z*2^{1/2}*\left(Ln\left(1\;+\;C{V_{a}}^{2}\right)+\;Ln{\left(1\;+\;CVw^{2}\right)}^{1/2}-\;1\right)\right] \end{array}$$

As the increase in SCr level (unidirectional) was evaluated, Z coefficient with 95% probability (*P *< .05) was 1.64.

Based on the pretreatment SCr levels of the patients individually, percent change on days 3 and 7 were compared with RCV. Results exceeding RCV increase value were considered significant.

## Results

Median age of the 47 patients included in the study was 74 (60–84) years and 32 of them were male. The indication to start administering colistin was ventilator-associated pneumonia in 37 of the patients, 3 had blood circulation infections, 4 had urinary system infection, and 3 had surgical site infections. There were 11 patients with pretreatment SCr levels exceeding the upper reference limit, 16 patients within the reference interval, and 20 patients below the lower reference limit.

**Figure 1.**
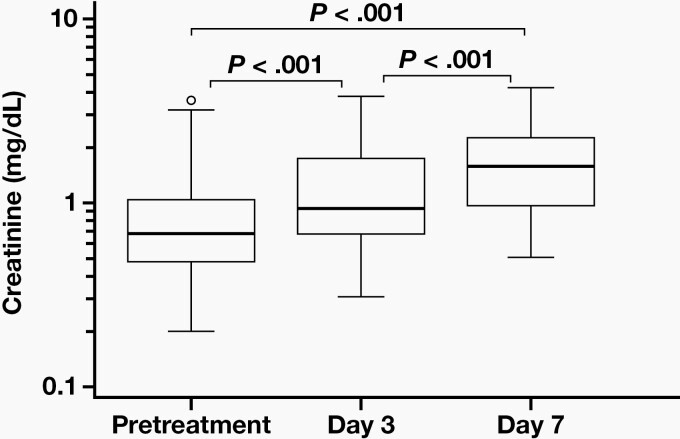
SCr levels at pretreatment, after dose on day 3, and after dose on day 7 in patients undergoing treatment with colistin.

When pretreatment SCr levels were compared with day 3 and day 7 levels after treatment, the increase in SCr levels was found to be statistically significant (*P *< .001). Also, there was a statistically significant difference between SCr levels at day 3 and day 7 (*P *< .001) (**Figure 1**). The number of patients developing nephrotoxicity after colistin treatment was 22 of 47 on day 3 and 35 of 47 on day 7.

According to data obtained from the EFLM biological variation database, for SCr, CV_W_ was 4.4 and CV_G_ was 14.1. Mean, SD, and CV values calculated for SCr are summarized in **Table 1**. The RCV was calculated as 15.6%.

**Table 1.** Calculation of the Within-Laboratory Precision for Serum Creatinine

Sample	Mean (mg/dL)	Repeatability		Within-Laboratory Precision	
		SD	CV	SD	CV
Precicontrol 1 (lot No. 41011000)	1.05	0.01	1.0%	0.03	2.5%
Precicontrol 2 (lot No. 41003200)	3.97	0.05	1.1%	0.14	3.6%

*CV, coefficient of variation; SD, standard deviation.*

When the percent increase in SCr levels after treatment was compared with pretreatment SCr levels, on day 3 there were 32 patients whose values exceeded RCV and on day 7 there were 36. RCV had 100% sensitivity and 60% specificity on day 3 and 97.1% sensitivity and 83.3% specificity on day 7 for colistin nephrotoxicity. Of the 47 patients, 10 with a false-positive rate on day 3 progressed to colistin nephrotoxicity on day 7.

**Figure 2.**
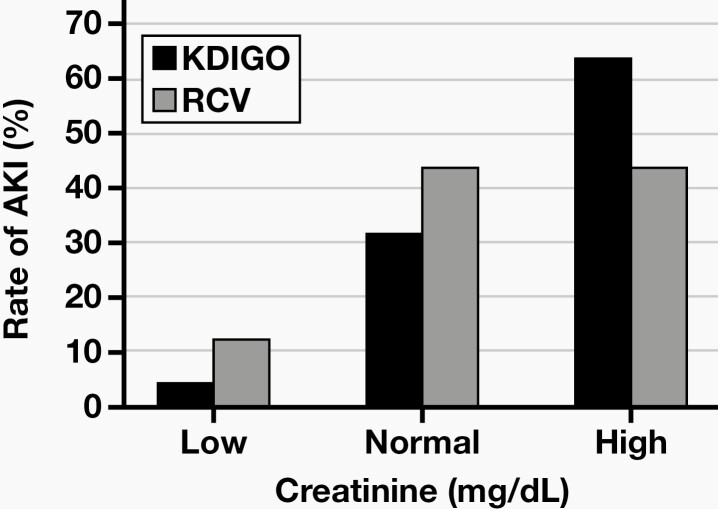
The rate of acute kidney injury (AKI) according to the reference interval on day 3. KDIGO, Kidney Disease: Improving Global Outcomes; RCV, reference change value.

**Figure 3.**
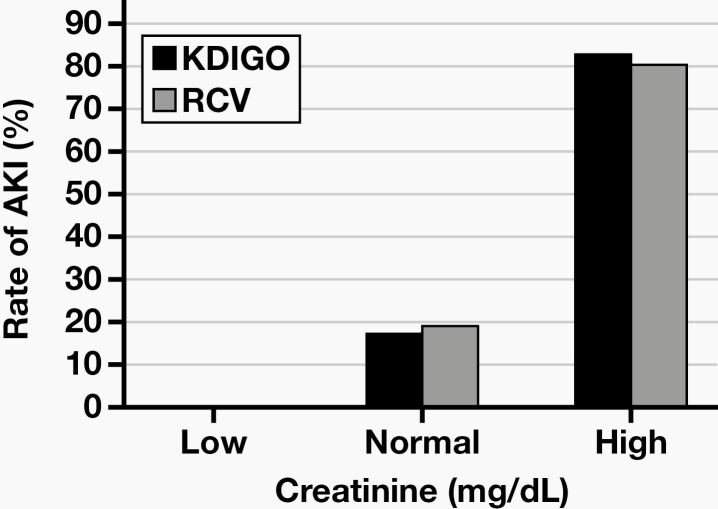
The rate of acute kidney injury (AKI) according to the reference interval on day 7. KDIGO, Kidney Disease: Improving Global Outcomes; RCV, reference change value.

On day 3, the number of patients with SCr values exceeding the upper reference limit and developing AKI according to the RCV and KDIGO was 14 and 16, respectively, whereas the SCr values were within or below the reference interval, although AKI developed in 18 patients according to RCV and 21 patients according to KDIGO. There were 28 patients with SCr levels exceeding both the RCV and the upper reference limit on day 7, whereas the SCr levels of 8 patients were within or below the reference interval although they exceeded the RCV. In a similar manner, although 30 patients exceeded the upper reference limit and were considered as having AKI according to KDIGO, the SCr of 7 patients with AKI did not exceed the upper reference limit on day 7. The distribution of patients developing AKI according to the reference interval is given in  **Figures 2** and **3**.

Patient evaluation by using RCV is given in **Table 2** as an example.

**Table 2.** Example of the Interpretation of the Creatinine Levels Using RCV

		Patient 1	Patient 2	Patient 3	Patient 4
Predose	mg/dL	0.78	0.65	0.56	0.64
After dose on day 3	mg/dL	1.79	0.89	0.45	0.66
	Change (%)^*a*^	129.49	36.92	−19.64	3.13
After dose on day 7	mg/dL	2.37	2.23	0.52	1.41
	Change (%)^*b*^	203.85	243.08	−7.14	120.31
RCV^*c*^	3rd day	15.6<129.49^*d*^	15.6<36.92^*d*^	—^*e*^	15.6>3.13^*f*^
	7th day	15.6<203.85^*d*^	15.6<243.08^*d*^	—^*e*^	15.6<120.31^*f*^

*RCV, reference change value.*

^*a*^ *(creatinine*_*after dose 3rd day −*_ *creatinine*_*predose*_ *)/creatinine*_*predose*_ *× 100.*

^*b*^ *(creatinine*_*after dose 7th day −*_ *creatinine*_*predose*_ *)/creatinine*_*predose*_ *× 100.*

^*c*^ *Reference change value, calculated as 15.6%.*

^*d*^ *The percent change in creatinine on 3rd* *day and 7th* *day exceeded RCV and the change is significant.*

^*e*^ *As the increase in creatinine level (unidirectional) was evaluated, the change is insignificant on 3rd* *day and 7th* *day.*

^*f*^ *The percent change in creatinine predose on 3rd* *day is below the RCV and the change is insignificant but the percent change in creatinine on 7th* *day exceeded RCV and the change is significant.*

## Discussion

In this study, serial SCr measurements were evaluated with RCV to show the development of nephrotoxicity in patients using colistin treatment. First, our laboratory’s RCV value for SCr was calculated, and for SCr unidirectional *P *< .05, we found it to be 15.6%. In a study where CVw was obtained from the Westgard website, SCr unidirectional RCV *P *< .05 was calculated as 15.03%.^16^ Reinhard et al^17^ conducted a study on 20 healthy volunteers and 19 patients with impaired renal function. For SCr, CVw of healthy volunteers was 4.7% and RCV was 13.6%; for patients with impaired renal function, CVw was 8.9% and RCV was 25%. In another study on CKD patients, SCr CVw was reported as 5.7% and RCV as 17%.^18^ The difference in RCV values is due to the within-laboratory precision, the use of different RCV formulas and Z coefficients, and obtaining CVW from previously published studies in the literature or from samples from healthy volunteers as well as a specific patient population.

We evaluated the SCr levels of patients receiving colistin treatment and analyzed their SCr levels before treatment and on days 3 and 7 after treatment. On day 3 and 7 after treatment, SCr levels were found to be significantly high compared with pretreatment levels. We found the risk for developing nephrotoxicity as 22 of 47 on day 3 and 35/47 on day 7. Hartzell et al^19^ evaluated 66 patients who had received colistin treatment for at least 3 days in their study, showing that 30 of 66 patients developed nephrotoxicity. In another study on patients receiving colistin treatment, 53.5% of the patients developed colistin-associated kidney damage.^20^ In a study evaluating 30 adult patients receiving intravenous colistin, 10 of 30 patients were found to develop nephrotoxicity during the first 5 days of treatment.^21^ In a case-control study comparing aerosolized and IV forms of colistin for the treatment of ventilator-associated pneumonia, the risk of developing nephrotoxicity was found to be 50% for both forms.^22^ Spapen et al^11^ conducted a review on the side effects of colistin, stating that the risk for developing nephrotoxicity changed from 0% to 53.5%. We think that different rates are found due to differences in patient groups in the conducted studies, presence or absence of a history of renal dysfunction, differences in age, sex, obesity, underlying diseases, other medications in use, or different criteria in use. Despite the fact that different criteria were used, the studies all make classifications based on the changes in SCr levels.

In our study, when day 3 SCr levels were compared with basal SCr levels, the ratio of patients with percent increases that exceeded the RCV was 32 of 47, whereas on day 7 this figure was 36 of 47. Nephrotoxicity developed on day 7 in 9 of 10 patients with a false-positive rate on day 3. Therefore, RCV can provide a warning for possible nephrotoxicity before SCr reaches the identified level by monitoring patient results on a daily basis. The second patient in **Table 2** can be given as an example of this situation. Before colistin treatment, SCr was 0.65 mg/dL, and SCr on day 3 was measured as 0.89 mg/dL. As the increase in the patient’s SCr did not exceed 50% of the basal level and there was a lack of increase of 0.3 mg/dL in SCr on day 3, this patient was evaluated as not having developed nephrotoxicity. However, when SCr increase on day 3 was compared with the RCV, the increase in this patient’s result was significant. On day 7, the patient’s SCr was increased to 2.23 mg/dL showing developing nephrotoxicity; the increase in SCr was significant when compared with RCV as well.

Patients are expected to show an increase in SCr due to the use of nephrotoxic agents; however, SCr might remain within the reference interval, leading to mistaken interpretation.^23^ In many previous biological variation studies, the index of individuality of SCr was shown to be <0.6.^24–26^ It is recommended to use the percentage of the change from baseline or in serial measurements.^27^ In this study, if SCr was only evaluated according to the reference interval, 18 of 47 patients on day 3 and 8 on 47 patients on day 7 who were diagnosed AKI using the RCV would be incorrectly considered non-AKI.

One of the limitations of this study was the inaccessibility of the records of preanalytical factors interfering with SCr, as it was designed as a retrospective study. In addition, although persons with CKD were included in the study, the value of RCV could not be shown in patients above the upper reference limit because of an insufficient number of patients. Although RCV was compatible with KDIGO in 10 patients with SCr levels exceeding the upper reference limit before treatment, AKI could not be detected in 1 patient with an SCr level that was approximately 3.3-fold the upper reference limit with RCV on day 3 nor on day 7. A study with a larger patient group is needed to demonstrate the diagnostic accuracy of RCV in the patients that developed AKI with underlying CKD.

## Conclusion

This study provided an example of the clinical use of RCV. As shown in the results, small but significant changes in SCr level can be detected with RCV. Even if this increases the false positivity rate, it will provide early detection of the development of AKI, especially by follow-up assessment of SCr levels caused by treatments such as colistin that have nephrotoxic side effects. We think including RCV using KDIGO criteria would be a more accurate and faster approach for decision making.

## Abbreviations

SCr: serum creatinine
RCV: reference change value
CVw: intra-individual biological variation
CVG: inter-individual biological variation
EFLM: European Federation of Clinical Chemistry and Laboratory Medicine
RIFLE: Risk, Injury, Failure, Lost, End Stage Renal Disease
AKIN: Acute Kidney Injury Network
KDIGO: Kidney Disease: Improving Global Outcomes
AKI: acute kidney injury
IV: intravenously
SD: standard deviation
CLSI: Clinical and Laboratory Standards Institute
CKD: chronic kidney disease
